# Analysis of Healthcare Professionals’ and Institutions’ Roles in Twitter Colostomy Information

**DOI:** 10.3390/healthcare11020215

**Published:** 2023-01-11

**Authors:** Pedro Jesús Jiménez-Hidalgo, Beatriz Jiménez-Gómez, Carlos Ruiz-Núñez, Sergio Segado-Fernández, Fernando Diez-Villacañas, Fidel López-Espuela, Ivan Herrera-Peco

**Affiliations:** 1 Traumatology and Orthopedic Surgery Service, Hospital Universitario Nuestra Señora de Candelaria, Ctra. Gral. del Rosario, 145, 38010 Santa Cruz de Tenerife, Spain; 2 Department of Nursing, Human Nutrition and Dietetics, Universidad Europea de Madrid, Calle Tajo, s/n, Villaviciosa de Odón, 28670 Madrid, Spain; 3 Ph.D. Student Program in Biomedicine, Translational Research and New Health Technologies, School of Medicine, University of Malaga, Blvr. Louis Pasteur, 29010 Málaga, Spain; 4 Department of Health Sciences, Universidad Europea de Canarias, Calle Inocencio García, 1, La Orotava, 38300 Santa Cruz de Tenerife, Spain; 5 Faculty of Health Sciences, Alfonso X el Sabio University, Avda Universidad, 1, Villanueva de la Cañada, 28691 Madrid, Spain; 6 Metabolic Bone Diseases Research Group, Nursing Department, Nursing and Occupational Therapy College, University of Extremadura, 10003 Cáceres, Spain

**Keywords:** colostomy, disinformation, health communication, healthcare institutions, nurses, public health, social media

## Abstract

Social media represents a powerful tool for disseminating verified health information on topics such as colostomy, and the roles of healthcare professionals and institutions to ensure the veracity of the information conveyed is increasingly relevant. The main objectives of this study were to analyze the roles of these healthcare professionals and institutions in the conversation about colostomy, without being framed in a specific health communication campaign, and to know the use of reliable information in the conversation. The study was carried out by analyzing Twitter messages containing the hashtag “colostomy” and “Chron” between the 1 January and the 30 April 2022. It was conducted using the NodeXL software, focusing on content analysis of tweets and users’ accounts. The results show that accounts with healthcare activity influence the impressions generated on the network (*p* = 0.018), finding that nurses are the most active healthcare professionals (22.24%) also having a significant effect on the overall network interactions (*p* = 0.022). In contrast, we found that institutions do not actively participate on the network. We emphasize the responsibility of institutions for health education and the need for professionals to improve communication skills on social networks, but also the need to improve communication skills on social media to support public health campaigns through these increasingly important channels.

## 1. Introduction

Colorectal cancer (CRC) and inflammatory bowel diseases (IBD) are the pathologies that most frequently require the performance of an intestinal stoma. CRC ranks first in incidences of these techniques, and it is the second leading cause of death in Europe in both men and women, with 446,000 new cases diagnosed each year [1].

Approximately 100,000 people undergo colostomy surgery each year [2]. In Spain, 16,000 new patients per year are colostomy carriers. According to these data, the approximate number of people with a colostomy in Spain exceeds 70,000 cases, and this figure continues to rise [3].

The utilization of a colostomy increases survival rates in these patients; however, there is a risk of postoperative complications after colostomy creation [2,4]. These complications can be classified as early or late, depending on the time of onset. Early complications are those that occur within 30 days after surgery. The most common are peristomal irritant dermatitis, mucocutaneous separation, retraction, and tissue necrosis. Late complications occur after this postoperative period and may include peristomal hernia, prolapse, stricture, and granuloma [2]. The prevalence of complications varies from 10% to 70% [2,5,6]. In addition, ostomized patients face different physical and functional losses—as well as psychological, emotional, and social repercussions—that directly affect their quality of life [1,7].

The performance of a stoma involves a drastic alteration of body image, loss of voluntary control of stool elimination, and the need to use a bag to store fecal materials discharged from the bowel [7]. The preoperative and postoperative care that these patients receive improves their acceptance of their condition, so it is very important to know the educational needs of patients and caregivers [8,9,10,11]. Proper stoma care can reduce the occurrence of complications, hospital readmission rates, and the economic cost to healthcare institutions and services, improving the quality of life of these patients [11].

Changes in lifestyle can be very challenging for patients with a new colostomy and require teaching skills and providing a lot of support. It is important to include a family member or caregiver in this process and to support the patient in acceptance and adjustment. Patients and caregivers should be provided with information about the outcome of the stoma and any complications that may develop [12].

Nursing professionals play a very important role in the care of these patients because the management of colostomies and their complications is a nursing function, and as mentioned above, this care focuses on physical, social, and emotional support [8,9,10,11].

However, these patients, once they return home, can be immersed in a multitude of doubts and uncertainty about their care or their disease, and in most cases, they do not have a nurse on site who can resolve these doubts. Thirty-five percent of patients with stomas from public hospitals do not have access to specialized colostomy care. This figure is even higher if they come from private centers [3]. These patients seek to resolve their doubts with the easiest and most accessible tool they can have, the internet.

Social networks have become a very powerful tool when searching for health-related information or collecting such information [13,14,15], but is this information correct? Is it based on scientific evidence? Are healthcare professionals, specifically nurses, the ones who generate health information regarding ostomies and their care?

The body of health information underlines the importance of effective scientific communication [16]. The main barrier to health information is the lack of quality and reliability. The interactive nature of social media magnifies these problems, as any user can create content on a website. Social media users may also be vulnerable to both hidden and open conflicts of interest that they may be unable to interpret [17].

Health information, also known as “health literacy”, refers to the information that each individual needs to make good health decisions [18].

The media are powerful channels for the dissemination of health education and information. They also contribute substantially to health awareness and promotion, making them an essential mediator for health communication. They play an important role in changing attitudes and intentions, and in influencing health-related behavior. “Health communication” is a broad term, defined as the study or use of communication techniques to improve the health sector. The effectiveness of media in health communication lies in strong written, verbal, and visual communication strategies that can influence public opinions and perceptions [19].

Social media are an immensely powerful source of social influence, with the ability to help people express opinions on issues that matter to them or to alter attitudes and perceptions about situations and problems [20].

The search for health information on social networks is becoming more frequent [21] and is an important element in the health literacy of the population. Because of this, it is necessary that health professionals and health institutions and centers become referents offering truthful and reliable information to any user who accesses these social networks in search of health information [13].

Social networks provide tools for sharing information, discussing healthcare policies and practices, promoting healthy behaviors, and interacting with the public, as well as educating and socializing with patients, caregivers, students, and healthcare professionals, among others [17,22,23]. Social networks allow rapid dissemination of information due to the high number of users they have. Examples of this are the users that we can find on Twitter (544 million), YouTube (2.51 billion), and Instagram (1.38 billion) [24]. The number of people who can be reached by health information allows us to define that an adequate communication strategy can help to achieve the objectives of public health [17]. Thus, social media websites are becoming valuable research tools for patients, particularly in health care [14,15], and must begin to be integrated into public health objectives, using these tools to inform and educate the population, placing health professionals at the center of the response as key elements in the dissemination of truthful health information.

Therefore, physicians, nurses, and other healthcare professionals must place themselves at the center of responses as disseminators of truthful healthcare information and may need to compete for audience attention while positioning their messages in various media [25], directly countering disinformation and producing genuine information based on scientific evidence [26,27,28]. Twitter is a social network with high popularity among healthcare professionals, being used not only for personal use but also for professional use, creating awareness for health promotion campaigns, for instance [15,29]. Twitter can be perceived as a forum for communicating health information, better than other social networks such as Facebook, Instagram, or even YouTube, chosen by health professionals but also by health organizations [13,29]. 

In this context, the main objectives of the present study were (i) to analyze the roles of these healthcare professionals and institutions in the conversation about colostomy, and (ii) to know if the information posted on the social network Twitter is conveyed by healthcare professionals using scientific evidence or if, on the contrary, it does not have scientific value.

## 2. Materials and Methods

### 2.1. Study Design and Ethics

An observational, retrospective, time-limited study was proposed in which activity on the social network Twitter was analyzed.

Because this study was performed on a social network and only activity among Twitter users was measured, no approval from a Research Ethics Committee was required. However, the accounts of individual users were anonymized to develop good research practices on social media [30].

### 2.2. Data Collection

The information from the tweets was extracted through an API (application programming interface) search tool using the professional version of the software NodeXL (Social Media Research Foundation, Redwood City, CA, USA). To achieve the objectives proposed in this study, the Twitter users included in the data analysis were those who had sent tweets with the following features: (i) tweets published in Spanish; (ii) tweets containing keywords or hashtags “colostomy”, “colostomy”, and “Chron”; and (iii) tweets posted between 1 January 2022 (00:00 a.m. CET) and 30 April 2022 (23:59 p.m. CET).

### 2.3. Data Analysis

The analysis of the data compiled was performed in several steps. The first step was to analyze the most influential Twitter users who employed the analyzed hashtags, as well as their characteristics, using the betweenness centrality score (BCS), which measures the influence of a vertex over the flow of information to other vertices, always if information will travel through the shortest vertex path. The BCS value reflects how a user can control the information, choosing whether to share it or not and disclosing it to his network [20,25]. Secondly, we analyzed users’ activity to identify content, activities, and/or influential users that can be strongly associated with overall Twitter activity, measured by the metrics of interactions and impressions. The interactions were defined as “favorite” and “retweets”. Meanwhile, the impression is an indicator of the propagation of information, obtained when the number of tweets is multiplied by the number of followers [26]. 

Third, an analysis of both the users’ account descriptions and the contents of the tweets was performed. Users’ accounts were analyzed by searching their descriptions for identification as healthcare professionals. It is important to note that the categorization of healthcare professionals, as well as their possible specializations, was based on the description that users included in their public profiles.

The fourth step was performing a content analysis on the categories created after analyzing the data. It is important to note that, in this category analysis, only original tweets were considered because they were those that generated the actual content disseminated throughout the user network. The content and category coding were performed independently by two researchers and were corroborated by a third person whereby any differences in approach and focus were always discussed and resolved with full agreement.

Finally, for data analysis, descriptive and inferential statistics were used via the Statistical Package for the Social Sciences software (SPSS) version 23.0 (IBM, Armonk, NY, USA). First, the normality distribution of data was tested with Kolmogorov–Smirnov’s test and homoscedasticity with Levene’s test. The comparison between groups was performed with the U Mann–Whitney test. The statistical level of significance was set at *p* < 0.05.

## 3. Results

### 3.1. Network and User Analysis

With the data collected from the hashtag #colostomy, it was observed that a total of 281 Twitter users participated, amounting to 9603 interactions and 161,492,005 impressions. The network is composed of 809 messages, of which 123 (15.2%) were tweets (considered as original content), 223 (27.56%) retweets, 66 (8.16%) replies, and 400 (49.44%) mentions. 

Of these users, it was found that a total of 21 accounts (7.47%) were identified as official accounts of health institutions, whereas 76 accounts (27.05%) were identified as healthcare professionals. With respect to the accounts not linked to health activity, it was found that the vast majority, 153 accounts (54.45%), were cataloged as users, whereas 15 accounts (5.33%) corresponded to patient associations (See Table 1). It is important to note that no account was found that could be classified as an automated account or better known as a bot.

The number of tweets on the network linked to the different types of users shows that 5.69% (79) corresponded to colostomy patient associations, 2.44% (3) to health institutions, 9.76% (12) to healthcare professionals, and, finally, 56.1% (69) to companies. However, when analyzing the possible weight of the companies on the network, it was observed that it was not significant (*p* = 0.797) (see Table 1).

Regarding the impact of the accounts associated with healthcare activity, it was found that the number of impressions generated represented 1.72% of the total (2,774,432) and the interactions represented 24.66% (2368) with respect to the total.

It was observed that users associated with healthcare activity, institutions, and professionals had a significant weight in terms of interactions generated (*p* = 0.018). Regarding the impressions generated, users without healthcare activity generated a greater amount of interaction on the network compared to users with healthcare activity (*p* = 0.76) (see Table 1).

On the other hand, when analyzing the role of the healthcare professionals in the general colostomy network, it was found that they did not have any influence on the impressions (U = 7298; *p* = 0.451) but they had a significant influence on the interactions (U = 6405; *p* = 0.025). A similar situation was observed when analyzing the weight of the health institutions: they did not have an influence with respect to the interactions generated (U = 101; *p* = 0.417), although a positive effect on the impressions was observed (U = 231.5; *p* = 0.019).

**Figure 1:**
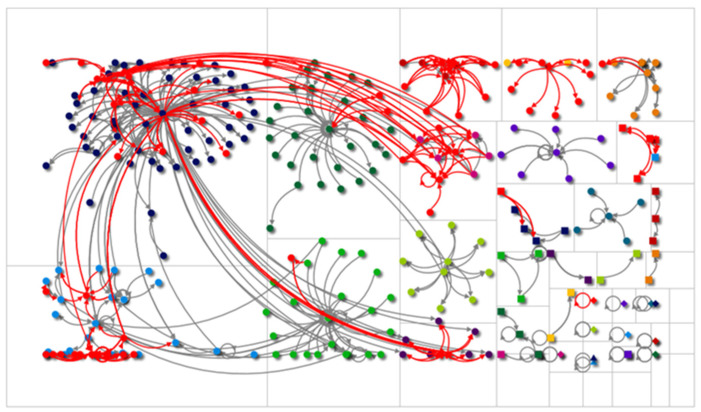
Representation of the colostomy network. The observed colors represent the different user groups that were identified in the network analysis. Furthermore, the red circles represent nurses present on the network. Red arrows represent communication with other users.

Going deeper into the role of nurses, the largest group, it is found that within the total network, they did not influence the impressions of the network (U = 6432; *p* = 0.399), although a significant influence was found in the interactions generated by the nurses (U = 5252; *p* = 0.003) (See Figure 1). With respect to the effect of nurses within the users with healthcare activity, nurses exerted a significant influence both in the impressions generated (U = 606; 0.001) and the interactions (U = 887; *p* = 0.0057). Finally, nurses, when compared with other healthcare professionals, were found to have a significant effect on interactions (U = 224; *p* = 0.022).

When analyzing the specific weights of each of the main groups of users found on the network who do not have healthcare-related activity, it is worth highlighting the role of companies. Within the network of users not performing a clinical activity, it was found that companies have an important role, contributing with a significant weight in the impressions (U = 528; *p* = 0.013) and interactions (U =4 86; *p* = 0.008). However, the influence of the companies with respect to the network total was not significant, neither in impressions (U = 1038; *p* = 0.826) nor in interactions (U = 935; *p* = 0.499). Likewise, the members of the network identified as users showed a significant influence on impressions compared with the general network (U = 6593; *p* = 0.001), although this did not happen when analyzing interactions (U = 9049; *p* = 0.272). When assessing the effect of users within the group of non-clinical activity, it was found that they did have a significant effect on impressions (U = 1733; *p* = 0.001) but not on interactions (U = 2706; *p* = 0.34).

### 3.2. Influence of Users Having Healthcare Roles and Content Analysis

The most influential users in the colostomy network were analyzed and categorized using the BCS (Table 2). It was found that within the 10 most influential users there are 3 accounts associated with healthcare activity: two nurses (Co4 and Co7) as well as a health institution (Co8). It should also be noted that the patient associations stand out as elements with a great capacity for disseminating information, which can be seen by the finding of 3 accounts (Co3, Co5, and Co9) among the 10 most important. Finally, and as a noteworthy fact in the colostomy network analyzed, the account which has the highest BCS value is a company that sells products aimed at colostomy patients. 

Finally, an analysis of the messages sent by health institutions and professionals was undertaken, more specifically nurses, as they were the users who had the most significant influence on the interactions generated in the colostomy network.

In relation to health institutions, it was found that in some cases the messages were just communicating that healthcare professionals who work in these institutions are trained to offer attention and care for patients. In other cases, it was observed that the institutions spread the message of ostomized patients’ initiatives to normalize colostomy (Table 3). 

Next, the messages sent from nurses present on the network were analyzed: a total of 179 messages included replies, mentions, retweets, and tweets. It was found that 42.46% (76) had no links to sources confirming the health-related information they provided in their tweet, 27.37% (49) were related to a company oriented to produce accessories for colostomy patients, 17.32% (31) referred to patients’ own experiences, and 6.15% (11) promoted nurses’ private courses focused on recommending colostomy care for patients. In relation to the analysis of the perceived credibility of the messages sent by nurses on this network, it was found that 6.7% (12) shared information from sources based on scientific evidence and provided links to the appropriate scientific and technical documents. Examples of these types of messages can be found in Table 3.

## 4. Discussion

This study analyzed the roles of healthcare professionals and institutions in the generation and dissemination of information and content on the social network Twitter, studying the behavior of a network built around a conversation about colostomy. The objective was not only to analyze the participation in a specific moment around a specific campaign but also the effective presence of health-related users such as healthcare professionals and institutions, to ensure that the health information given in that conversation was reliable. In the present study, it was found that those accounts having a clear healthcare approach do not have significant weight with regard to interactions generated by the information sent by healthcare professionals. This contrasts with what has been previously described in the literature [13,31,32]. However, it was observed that the interactions generated by these accounts were not important in the total volume of the network, something previously described for campaigns focused on social networks in Spain, such as those for influenza vaccination [33] or vaccination against COVID-19 [31,32].

On the other hand, when individually assessing the roles of health institutions and healthcare professionals on the network, it is found that institutions have a significant effect on impressions because they are accounts that represent health organizations and have a high number of followers [32,33,34]. Nevertheless, when analyzing interactions, no effect on the network was observed, corresponding with the usual behavior of these accounts, which generate information but not interactions with users [22,31]. The high impact of institutions on the impressions generated contrasts with previous findings by other authors, who state that healthcare institutions have an insignificant presence and are not considered central elements for the dissemination of information in campaigns or conversations guided by specific hashtags [35].

We also found a high presence of healthcare professionals on the network, in agreement with what has already been stated by other authors who also concluded that their presence is growing on social media [17,36,37]. A growing tendency was reinforced since the onset of the COVID-19 pandemic in 2020 [38,39]. It also was found that the weight of healthcare professionals in the analyzed network was significant with regard to the interactions generated. This situation may be associated with the role that healthcare professionals have traditionally played in everything related to supporting individuals and communities in understanding messages related to healthcare [40] and their ability to establish conversations with the population to resolve the doubts raised [41].

It should also be noted that healthcare professionals are considered influencers on health issues [31] in addition to their traditional consideration as figures who help to understand information [38] that improves self-care in patients [38]. This capacity of healthcare professionals, more specifically nurses [17], may explain the significant influence in the colostomy network when it comes to generating conversation and interactions with other users. Nurses generate the greatest number of interactions both at a general network level and among the rest of the healthcare professionals. This is corroborated by the existence of two nurses among the users who transmit the greatest amount of information in the network [20].

The analysis of the general user’s role shows that they influence the general network, specifically impressions, coinciding with what has been previously stated by some authors in other conversations about health issues on Twitter [32,35]. However, in users with no healthcare activity, the focus of companies on products for ostomates stands out. It is found that although they do not have a significant influence on the overall network, they do on non-healthcare users. This presence may be related to the alignment of companies in health initiatives [42] following “marketing washing” techniques such as those already seen in food [42] or breastfeeding [43]. However, the fact that numerous healthcare professionals and users take these messages to be re-disseminated in their networks may be related to the scarcity of original content generated by health institutions or healthcare professionals [13,31], causing this content to be widely disseminated.

When analyzing the nature of the messages sent on the network by institutions and healthcare professionals, more specifically nurses, it can be found that much of the activity carried out by them is of a personal nature, something that is aligned with what has been described by numerous studies [37]. About the activity when disseminating health information related to colostomy, it was observed that the information provided by these healthcare professionals did not contain links to scientific articles or technical documents, causing nurses not to be the main generators of verified information [31] and leaving that space to other types of users who could disseminate unverified information. This situation, in health information management, may have an influence on making better decisions about ostomized patient care [44]. 

On the other hand, in the case of healthcare professionals and institutions, it is found that they disseminate messages from patients describing personal life experiences, enhancing the visibility and normalization of colostomy [45], although they are messages with emotional content and do not provide verified health information. 

Finally, it should be noted that this study has several limitations, mainly related to the design, which was cross-sectional, and, secondly, the participant selection criteria, having used only Twitter to detect users as potential participants. Likewise, we would like to point out that Twitter, being a social network, only reflects the participation in conversations about the health of those defined users who, although they are health professionals, wish to participate out of personal interest. Furthermore, retrieving information using a specific hashtag and keywords may have missed users who posted messages without using these keywords. 

## 5. Conclusions

The results shown in this study allow us to know the typology of messages on colostomy, highlighting situations of interest such as the low proportion of messages with health information that was not supported by any type of scientific evidence. It was observed that there is a high number of messages with a focus associated with testimonials or personal experiences. This situation is not unusual on social networks, as has been described in numerous previous studies, because social networks continue to be used with a more personal than professional focus and this influences the way of communicating through them. It is of particular interest to note that messages with health information, without providing external links to documents that support the information provided, are one of the reasons that many authors are highlighted as an essential element for the reliability of the message to be questioned.

It is critical to develop and implement public health surveillance programs including social media monitoring as a priority action, as we have seen when conducting an analysis of the conversation outside the scope of an international colostomy-specific campaign day. Creating “observatories” for assessing social media conversations on public health issues will allow for a rapid response to disinformation. The later it is implemented, the more complex it will be to get across the correct information to the population. However, these actions must be led by health organizations, both public and private. 

Surveillance programs should also include communication actions aiming to create and disseminate verified health-related content in appropriate and understandable formats for the population to counteract the disinformation generated. From our point of view, it is necessary for health organizations to be present to monitor potentially dangerous hashtags, providing an accurate discourse and a reliable source of healthcare information. While it is paramount that public institutions are present and lead these campaigns, we believe it is very important that these institutions be present on social networks, given that they are an increasingly relevant vehicle for the transmission of health information. Likewise, this presence cannot be associated only with occasional participation, but it would be necessary to establish working procedures that make health information understandable and easily accessible to the entire population. Of course, it is of special interest to apply a lesson learned during the COVID-19 pandemic, which is that public institutions should offer consistent information and not modify the messages and information offered to the population, because these changes may generate mistrust in a part of the population to whom the message may be addressed. This is where healthcare professionals, as well as individual users, can act as reference figures to whom users of social media can turn to obtain health information and, of course, combat health disinformation.

In the current situation, where an increasing percentage of the population uses social networks to access health information, we consider that coordinated health communication actions on social networks are a necessity. The agents that should participate in these actions would be the health professionals themselves because they are the ones to whom a large part of the population confers great authority on health issues. However, institutions should also develop communications actions, probably based on the participation of health professionals themselves, but without forgetting the participation of citizens who can serve as agents for the dissemination of reliable information generated by health institutions and professionals. The focus of these actions would be to develop training actions that encourage health professionals present in social networks to use them as an extension of their clinical activity and thus collaborate in the dissemination of verified health information, becoming points of reference for users of social networks. For this purpose, it is considered necessary to implement training actions for healthcare professionals, even citizens, in the use of social networks to enhance their participation and improve the effectiveness of communication. It is important to focus these actions on showing how to prepare reliable tweets, emphasizing the veracity of the content by attaching external and reliable sources, and being careful when writing tweets.

## Figures and Tables

**Table 1:** Users’ characteristics on colostomy network.

	Description	N (%)			Impressions		Interactions	
					Mean	U; *p*-Value	Mean	U; *p*-Value
Users	Healthcare	100 (35.58)			23.68	5559; 0.018 *	27,744.32	6677; 0.76
	Non-healthcare	181 (64.42)			40.19		881,764.29	
Users having a healthcare role	Healthcare institutions	24 (8.54)			19.66	294; 0.001 *	78,750.42	846; 0.597
	Healthcare professionals	76 (27.05)			24.95		11,637.13	
			Nurses	63 (22.42)	27.92	350; 0.633	13,098.5	224; 0.022 *
			Physicians	8 (2.85)	13.87	178; 0.258	5852.71	180; 0.268
			Nutritionist	3 (1.07)	3.66	64; 0.230	387	40; 0.670
			Others	2 (0.71)	6.9	1; 1.000	1994	1; 1.000
Users without healthcare role	Patient associations	15 (5.34)			15.2	1087; 0.438	3496	1154; 0.667
	Colostomy products company	9 (3.2)			176.62	528; 0.013 *	121,233	486; 0.08
	University	4 (1.42)			17.75	241; 0.053	116,212.25	361; 0.234
	Users	153 (54.45)			39.7	1733; 0.001 *	15,422.41	2706; 0.340

Note: The group “Others” in healthcare professionals includes 1 psychologist and 1 pharmacist. * *p* < 0.05.

**Table 2:** Characteristics of most influential users in the “colostomy” network.

Network	User Code	Description	BCS	Network Activity
Colostomy Network	Co1	Company	18,716.711	Account associated with a company marketing accessories for colostomy patients.
	Co2	Citizen	5114.805	Personal experiences of a colostomy patient.
	Co3	Patient association	4944.939	To offer information to society and relatives of ostomized patients.
	Co4	Nurse	4,841,319	Mixed-use account: personal and professional activity. Focused on patient care.
	Co5	Patient association	1587.533	To offer information to society and relatives of ostomized patients.
	Co6	Citizen	1438.613	Personal account. No defined use.
	Co7	Nurse	1,345,818	Account with a professional approach. Focused on patient care and development of the nursing profession.
	Co8	Health institution	1315.381	Hospital account.
	Co9	Patient association	1204.221	To offer information to society and relatives of ostomized patients.
	Co10	Citizen	1196.000	Personal account. No defined use.

Note: The code “Co1” means that we describe the user with rank 1, classified by the BCS, in the colostomy network.

**Table 3:** Examples of messages by health institutions and nurses in the colostomy network.

Original Source	Message	Message Function
Nurse	Save the date! 31 March. 17 h. Webinar with recommendations for the care of your colostomy, sign up!	Training course in care for ostomized patients. Taught by a health professional on a private basis.
Company	#Coloplast and the Madrid Ostomates Association claim greater visibility for ostomates, people who face the fear of a hypothetical social rejection and the difficulties involved in the #colostomy.	To raise awareness of the problems of ostomized patients. Company support for this initiative.
Citizen	The intervention of the #stomatotherapist nurse is key in the quality of life of the ostomate, according to the conclusions of the U&A study in #Colostomy.	Providing information on the role of nurses in care of ostomized patient care. Evidence-based source contribution.
Citizen (ostomized patient)	#ColostomyDay2022 The road to #colostomy has not been easy but thanks to “my guardian angel,” I now have a new life, explains @xxxxxxx	Colostomy patient testimonial.
Nurse	It is critical to remove excess hydrocolloid powders to ensure proper adhesiveness of colostomy devices. Via @xxxxxxx #docenciacampillo #colostomy #colostomy #colostomy #nursing #tcae	Providing information on colostomy management in ostomized patients. Not providing a technical or a scientific source for the assertion made.
Health institution	She is a young ostomate who flees from the frivolity of the networks to show herself as she is and to help people who, like for her, the colostomy was the only way to gain quality of life and escape from pain. She tells her story in the campaign “Anna and her tummy”.	Supporting the visibility of colostomy by sharing a message from an ostomized patient.
Health institution	Our Stomaterapeuta @ xxxxxxx will lead as IP a national multicenter study for the development of the map of the colostomy patient experience. Today she will present it together with @ xxxxxxx at #ColostomyDay2022 @GVADrPeset @GVAfisabio @GVAsanitat #investigacionenfermera	Dissemination of the scientific activity developed by nurses in a health institution. It does not provide useful information for patients in the colostomy network.
Health institution	Colostomy is a surgical procedure that consists, for colon cancer, in a small abdominal surgery to expel the intestinal contents into an airtight bag when the expulsion of stool or urine in a normal way cannot be done.	Message focused on spreading and explaining what colostomy is. The original message has a link to external information so that users can access more information.

Note: the ‘xxxxxxx’ means that we delete the user’s ID to avoid identification and to ensure the anonymization of individual users.

## Data Availability

The data that support the findings of this study are available from the corresponding author upon reasonable request.
